# Health equity and unintentional pediatric cannabis ingestion

**DOI:** 10.1186/s40621-026-00710-4

**Published:** 2026-09-04

**Authors:** Melissa P. Blumberg, Amy D. Thompson, Brandon George, Elizabeth Lendrum, Wendy J. Pomerantz

**Affiliations:** 1 Clinical Pediatrics, Department of Pediatrics, Department of Emergency Medicine, Children’s Hospital of Philadelphia, 3401 Civic Center Blvd, Philadelphia, PA 19104 USA; 2 Clinical Pediatrics, Division of Emergency Medicine, Nemours Children’s Hospital, Sydney Kimmel Medical College of Thomas Jefferson University, 1600 Rockland Road, Wilmington, DE 19806 USA; 3 Division of Biostatistics, College of Population Health, Thomas Jefferson University, 901 Walnut Street, Philadelphia, PA 19107 USA; 4 Division of Emergency Medicine, Comprehensive Children’s Injury Center, Department of Pediatrics, Cincinnati Children’s Hospital, University of Cincinnati College of Medicine, 3333 Burnet Avenue, ML #2008, Cincinnati, OH 45229 USA

**Keywords:** Cannabis, Equity, Ingestion, Social work, CPS, Social deprivation index

## Abstract

**Background:**

Unintentional pediatric cannabis ingestion has been rising following medical and recreational legalization. In this study, we aimed to examine the epidemiology of pediatric cannabis ingestion following legalization and to assess the impact of demographic and socioeconomic factors on equity of social management.

**Methods:**

A retrospective cohort study was conducted at two high-volume children’s hospitals with level 1 trauma centers in the United States of America. Emergency department records from June 2016 to September 2024 were reviewed for children aged 0–6 years with a positive urine drug screen for tetrahydrocannabinol. Dates included were inclusive of medical (5/2011, 6/2016) and recreational legalization (4/2023, 11/2023) of cannabis products within both respective sites. Data collected included demographics, home zip code (used to assign a deprivation index), Emergency Severity Index triage level, and disposition. Manual chart review assessed ingestion type, location, suspected source owner, and involvement of social work, child protective services, and safe disposition planning. Descriptive statistics were used to characterize the population, and linear and logistic regression were used to determine the relationship between deprivation index, population characteristics, and social management.

**Results:**

Among 266 cases, most children were under age 2 (58.3%), male (52.3%), non-Hispanic White (43.2%), English speaking (98.9%), and publicly insured (71.4%). Ingestions increased over time, with 51.8% occurring in the last two years (2023–2024). Edibles (51.1%) were the most common ingestion type, often belonging to a primary guardian (40.2%). Most cases were triaged as ESI 1or ESI 2 (83.5%), with 41.7% evaluated in a trauma bay. Hospital admission was common (82.0%), with 20.7% of admitted children requiring critical care. Social work (95.5%) and child protective services (80.1%) were involved in most cases. No relationship was found between deprivation index and social work consultation (*p* = 0.52), child protective service reporting (*p* = 0.41), discharge to a primary guardian (*p* = 0.26), or discharge to a primary residence (*p* = 0.144).

**Conclusions:**

The incidence of unintentional cannabis ingestion presenting to the pediatric emergency department is increasing. Findings suggest equitable management across demographic and socioeconomic strata, highlighting high acuity and significant toxicity at presentation.

## Introduction

The number of unintentional pediatric cannabis ingestions has increased significantly following the medical and recreational legalization of cannabis and cannabis products [1–4]. Ease of cannabis access, increasingly concentrated products, and a market shift to edible goods have correlated with rising frequency and morbidity of pediatric exposures [5, 6]. Like other toxic ingestions, unintentional cannabis ingestion in children may prompt consideration of social service consultation, yet there are limited studies evaluating health equity in management and disposition of these patients [2, 7, 8, 13]. 

Cannabis legalization has contributed to an increase in private access to commercial dispensaries, and recent research estimates 79% of Americans live in a county with at least one cannabis dispensary [9]. In states with legal use, there is an increasing prevalence of caregivers of children reporting presence of cannabis in the home, across nearly all sociodemographic groups [10, 11]. However, less than half of caregivers report storing cannabis in a manner that is both locked and hidden from children [12], even though most unintentional cannabis ingestions occur in private residences [2–4, 13]. Increasingly popular edible products [14] can appear to look like gummies, candies, or other treats appealing to young children; there is a rising incidence of edible products implicated in pediatric ingestions [13, 15, 16, 17]. In recent years, product potency has increased [18], which has been associated with increased toxicity in young children [19]. Despite regulatory efforts in some states to limit child-appealing packaging and standardize product dosing [20], the number of unintentional cannabis ingestions in children continues to rise [2, 16]. 

Health equity remains a critical issue throughout medicine and public health. In pediatrics, disparities are evident in social work (SW) and child protective services (CPS) engagement during medical evaluations [21–26]. Some research has focused on the intersection of health equity in social management (i.e. – SW consultation, CPS reporting, disposition planning) of unintentional pediatric ingestions. One study in the PED found CPS referrals after any type of unintentional ingestion were disproportionately higher in Hispanic patients [27]. Another showed 27% of ingestion cases were referred to SW for further safety assessments and 15.4% were reported to CPS [28]. In the aforementioned study, hospital admission, prescription medication ingestion, exposures involving recreational/illegal/illicit substances, and increasing psychosocial adversity (risk factors such as domestic violence in the home, caregiver unemployment, prior CPS involvement) were found to be significantly associated with referrals, while age, race, and insurance status were not [28]. Limited research has investigated the link between health equity and pediatric cannabis ingestion. Two single-center studies reported a 79% and 100% SW consult rate, respectively; these studies do not comment on factors associated with SW consultation [7, 8]. Other single-center studies found CPS reporting rates of 59% and 86% [2, 13]. CPS reports related to cannabis ingestion were more common in younger children, those without a caregiver disclosure of a suspected ingestion at initial presentation, and those requiring higher levels of care [13]. 

Despite these findings, gaps remain. The frequency and incidence of unintentional pediatric cannabis ingestion is on the rise but there is limited data characterizing demographic and socioeconomic risk factors related to cannabis ingestion in a post-medical and post-recreational legalization era. To our knowledge, no prior study has investigated SW and CPS reporting related to cannabis ingestion in a multicenter evaluation post-legalization. Finally, no prior study has investigated a social deprivation index, a measure of social deprivation based on zip code, in conjunction with equity in the management of pediatric unintentional cannabis ingestion.

## Objectives

We aimed to characterize the epidemiology of pediatric cannabis ingestion following legalization and to assess the impact of demographic and socioeconomic factors on equity of social management and disposition.

## Materials and methods

This multi-center retrospective cohort study was conducted at two high-volume, free-standing children’s hospitals with level 1 trauma centers. Data was obtained electronically from the pediatric emergency department (PED) electronic medical records (EMR) (EPIC Hyperspace Version 2023). Patient encounters were included for children age less than or equal to 6 years cared for between June 2016 and August 2024 at Nemours’s Children’s Hospital (NCH) in Delaware, USA and Cincinnati Children’s Hospital Medical Center (CCHMC) in Ohio, USA inclusive of both the main (Cincinnati, OH) and satellite (Liberty Township, OH) campuses. These dates were selected based on legalization laws in the states. In Ohio, cannabis was legalized for medical use on June 8, 2016 and recreational use on November 7, 2023. In Delaware, cannabis was legalized for medical use May 2011, decriminalized in June 2015, and legalized for recreational use on April 23, 2023. We aimed to examine the population following legalization; thus, the later of the medical legalization dates between the states was selected for the start of data collection. The age group was selected based on prior reporting on unintentional pediatric cannabis ingestion [13, 16], as children in this group appear to be at increased risk of unintentional ingestions [29]. 

Pediatric ED encounters were selected based on a positive urine drug screen (UDS) for tetrahydrocannabinol (THC), the active ingredient in cannabis on standard urine drug screens. Urine THC testing is highly sensitive and specific [30–32]. NCH utilizes MedTox Scan Drugs of Abuse Test System, a one-step immunochromographic test for rapid, qualitative detection of one or more drugs in human urine, with a THC detection cutoff value of 50 ng/mL. Positive results are reflexed to Mayo Clinical Labs, with a detection cut off value of 5 ng/mL using mass spectrometry. CCHMC utilizes in-house liquid chromatography with confirmatory mass spectrometry, with a THC detection cut off of 50 ng/mL for liquid chromatography results and 4ng/ml for mass spectrometry results. Both hospitals’ confirmatory testing takes over 24 h. Thus, patients were included based on an initial positive screen, even if confirmatory testing was negative, as it was felt that clinical decisions would be based on the initial result in the PED. Patients were excluded if they were admitted directly to the hospital without emergency department evaluation, if they had a medical prescription for cannabis, if history indicated the patient intentionally ingested the product, or if there were other documented concerns about abuse during the encounter. During the study period, there were no standard guidelines for medical or social management (SW consultation, CPS reporting) of unintentional ingestions at either hospital.

Data collected from the EMR included patient demographics (age, sex, self-reported race, self-reported ethnicity, preferred language, insurance type, state, and home zip code), hospital length of stay, triage code, chief complaint, and disposition (admit, discharge, other). Self-reported race and ethnicity included non-Hispanic White, non-Hispanic Black, Hispanic, or other/unknown. Other includes patients who identified as American Indian or Alaska native, Asian, Native Hawaiian or Other Pacific Islander, other (not specified), or patients listing two or more races. Any patient with a Hispanic ethnicity was classified as Hispanic, regardless of race, including cases where race was missing. Race and ethnicity were considered missing if ethnicity was missing or if race was missing and the reported ethnicity was non-Hispanic or Other. Insurance type options included commercial, public, self-pay, and other/unknown. A social deprivation index (SDI), a measure of socioeconomic status, calculated based on geocoded zip code data, was assigned to each patient. Six weighted census variables are used to calculate the deprivation index with the resultant range of 0–1; higher scores indicate greater deprivation [33–35]. Finally, chart review was completed manually by study investigators to determine whether a SW evaluation or CPS report was completed prior to disposition. In both institutions, a SW note is placed in EPIC for all patients receiving an evaluation and clearly indicates presence of absence of formal CPS reporting. SW evaluation was defined by a note placed in the chart, signed by licensed social worker. CPS reporting was defined as SW documentation of any formal report by the clinical provider in response to the encounter, regardless of timing of evaluation (such as prior to discharge vs. anticipated home evaluation). Researchers also assessed where the ingestion occurred (home, daycare, school, public place, other/unknown), the suspected ingestion type (edible, gummy, liquid, oil, other, unknown), and the suspected source owner (primary guardian, extended family, caregiver, other, unknown). Similarly, the charts were reviewed to determine the discharge safety person (primary guardian, other) and discharge safety location (primary residence, other), if applicable.

Descriptive statistics were used to characterize the population, including demographics, clinical characteristics, and social outcomes, reported as frequencies and percentages. To examine the relationship between sociodemographic factors and ingestions, a series of univariate regression models were used. Categorical variables were dummy coded using indicator variables with a designated reference category. Linear regression assessed the association between insurance status and SDI, as well as racial/ethnic background and insurance status. Binary logistic regression was performed to evaluate whether SDI or racial/ethnic background predicted social outcomes, including SW consultation, CPS reporting, and discharge disposition. All models were unadjusted with no additional covariates included. Beta coefficients (B) and 95% confidence intervals (CI) were reported for linear models, while odds ratios (OR) and 95% CIs were reported for logistic models. Statistical significance was defined as *p* < 0.05 for all tests. All statistical analyses were performed using IBM SPSS Statistics (Version 30).

## Results

**Fig. 1:**
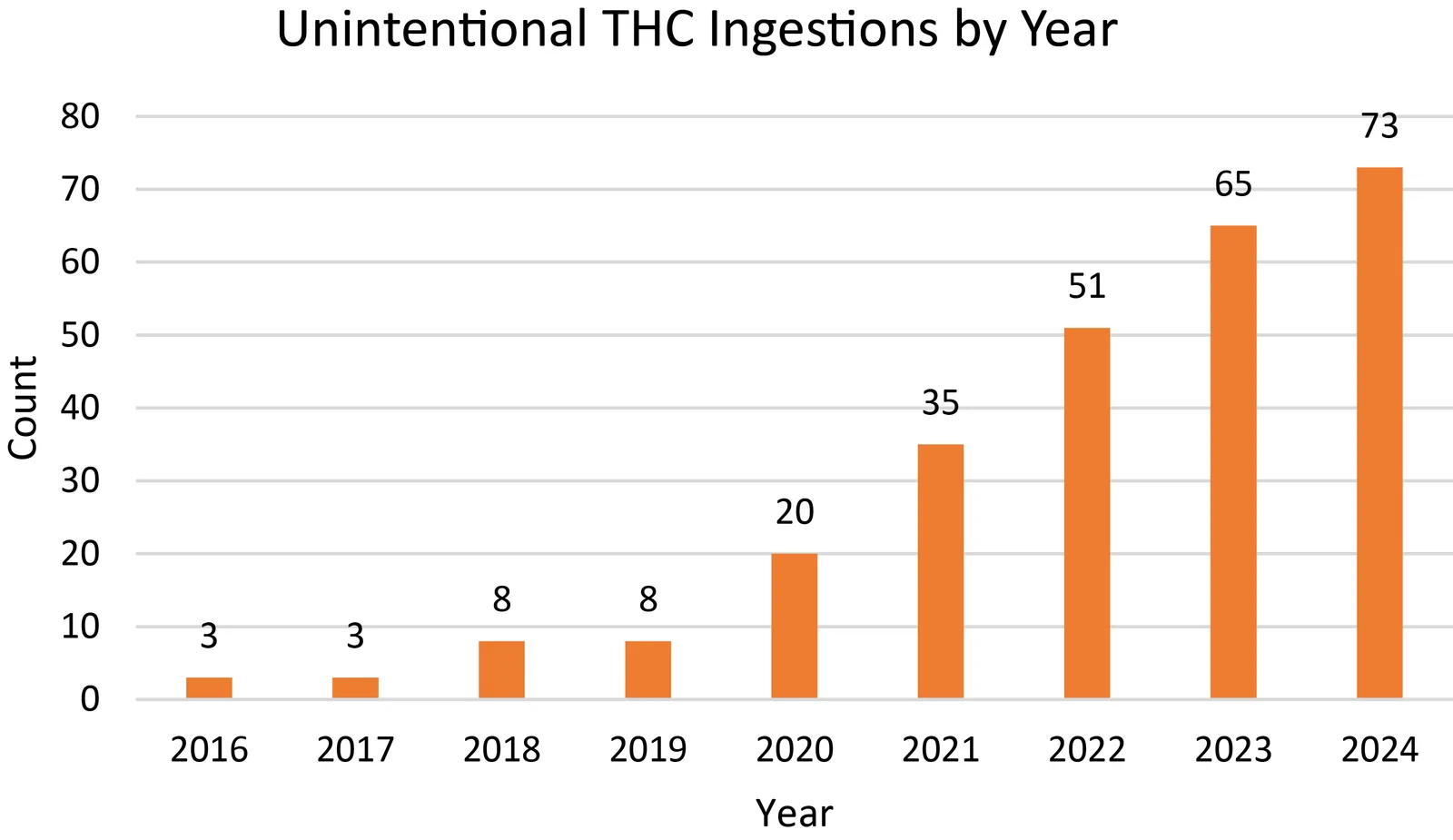
THC ingestion events from 2016–2024

There were a total of 266 patients included in the study, 77 from NCH and 189 from CCHMC (Table 1). These children were primarily less than age 2 (58.3%), male (52.3%), non-Hispanic White (43.2%), English speaking (98.9%), and publicly insured (71.4%) (Table 1). Comparatively, baseline demographic data of all ED visits to each site during the study period including NCH mean age 6, 52.7% male, 77.3% non-Hispanic, 50.1% White, 89.8% English speaking, 51.0% publicly insured, and CCH mean age 8.4 years, 50.7% male, 91.8% non-Hispanic, 61.0% White, 75.1% English speaking, and 59.0% publicly insured. The incidence of ingestions increased over the years of the study, with most occurring in the last two years between 2023 and 2024 (51.8%) (Fig. 1). The most common chief complaints were related to altered mental status (40.6%) and ingestion (37.6%) (Table 2). Most patients (83.5%) during initial PED triage evaluation were considered emergent, high-risk (ESI 2) or immediate, life-threatening (ESI 1), with 41.7% of patients evaluated in the trauma resuscitation bay (Table 2). All patients survived to discharge (100%). Most patients were admitted (82%), some requiring critical care (20.7%). Edibles (51.1%) belonging to a primary guardian (40.2%) were the most common implicated source of ingestion (Table 3). Over 95% of cases in the study had positive THC confirmatory testing. Most ingestions occurred in the primary residence (63.5%). SW (95.5%) and CPS (80.1%) were consulted in the majority of cases (Table 4). Most children were discharged with a primary guardian (82.6%) to their primary residence (85.9%).

In our study population, we found a relationship between insurance status and social deprivation index (*p* < 0.001); those with public insurance (B = 0.083, 95% CI 0.057–0.109) and unknown insurance (B = 0.053, 95% CI 0.006-0.100) were more socially deprived than those with commercial insurance. There is also a relationship between insurance and racial ethnic background (*p* < 0.001); compared to non-Hispanic White children, non-Hispanic Black children (B = 0.310, 95% CI 0.130–0.491) and Hispanic children (B = 0.386, 95% CI 0.021–0.750) were more likely to have public insurance. However, there was no relationship between SW reporting (OR = 0.12, 95% CI 0.00-74.43, *p* = 0.52) or CPS reporting (OR = 0.25, 95% CI 0.01–6.43, *p* = 0.41) with deprivation index. Similarly, there is no relationship between discharge home with a primary guardian (OR = 6.57, 95% CI 0.26–169.1, *p* = 0.26) or to a primary residence (OR = 13.562, 95% CI 0.409–449.58, *p* = 0.144) and deprivation index. There was no difference between SW consultation and site location (X^2^(1, *N* = 266) = 0.988, *p* = 0.320). Racial ethnic background was not a significant predictor of social work consultation (*p* = 0.67); compared to non-Hispanic White patients, odds of consultation did not differ with comparison to patients who identified as non-Hispanic Black (OR = 0.46, 95% CI 0.12–1.84), Hispanic (OR = 1.10, 95% CI 0.13–9.63), or other/unknown patients (OR = 0.48, 95% CI 0.06–4.07). There was a difference between site location and CPS reporting (X^2^(1, *N* = 266) = 17.45, *p* < 0.001), with higher rates of reporting at NHC (96.1%) than CCHMC (73.5%). There was no association between racial ethnic background and CPS reporting (*p* = 0.693), with no difference odds of consultation for non-Hispanic Black (OR = 0.71, 95% CI 0.36–1.41), Hispanic (OR 1.31, 95% CI 0.38–4.47), or other/unknown patients (OR = 0.97, 95% CI 0.38–2.49) compared to non-Hispanic White patients. Similarly, there was no association between racial ethnic background and discharge home with a primary guardian (*p* = 0.697), with odds of non-Hispanic White patients showing no difference compared to non-Hispanic Black (OR = 1.143, 95% CI 0.559–2.338), Hispanic (OR = 0.821, 95% CI 0.17–3.950), or other/unknown (OR = 1.707, 95% CI 0.665–4.378). Finally, there was no relationship between racial ethnic background and discharge home to a primary residence (*p* = 0.814), with odds of non-Hispanic White patients showing no difference compared to non-Hispanic Black (OR = 0.616, 95% CI 0.568–2.603), Hispanic (OR = 0.467, 95% CI 0.057–3.812), or other/unknown (OR = 1.167, 95% CI 0.390–3.490).

## Discussion

In this multicenter cohort of young children presenting to the PED after unintentional cannabis ingestion, cases increased substantially over time, with most exposures occurring in children under 2 years of age and primarily involving edible products. Clinical acuity was high, with frequent triage assignment of ESI 1 and 2, substantial use of resuscitation resources, and high rates of hospital admission, including critical care. We found high rates of involvement of SW and frequent CPS reporting in our population; there was no association between deprivation index or racial ethnic background to SW or CPS engagement or disposition planning.

Our findings align with accumulating evidence that pediatric unintentional cannabis ingestions have increased in the post-legalization era and that edibles are a driver of pediatric exposures [2, 3, 6, 13, 36] Edible products are often packaged in ways that resemble conventional snacks and stored in homes where adult users are present, factors that likely amplify risk in young children who are in the oral exploration stage [11, 12, 15]. Regulatory steps such as child-resistant packaging and dosing standards may mitigate risk, yet data suggest these measures have not been sufficient to reverse the upward trajectory of exposures; notably, there are no federal laws regulating safe storage of cannabis products [18–20]. 

The high proportion of children triaged as ESI 1 or 2, frequent trauma bay activations, and substantial admission rates underscore the significant resource burden that unintentional cannabis places on pediatric emergency and inpatient systems. Although no standardized THC dose threshold for toxicity in children has been established, higher doses are associated with severe and prolonged toxicity [19]. it is reasonable to infer that younger children are at greater risk due to lower body weight and the potential to ingest large quantities of highly concentrated edible products. These findings underscore the significant clinical burden of pediatric unintentional cannabis ingestion and highlight the growing strain on pediatric healthcare systems as cannabis becomes more accessible nationwide.

Despite prior literature documenting disparities in SW and CPS involvement across pediatric evaluations and in ingestion cohorts (e.g., higher referral rates by race/ethnicity in some settings) [23, 27, 28], our multicenter analysis did not detect associations between deprivation index or race/ethnicity and SW or CPS activation or disposition outcomes. Instead, SW and CPS involvement appeared consistent across groups. In the absence of national standards for mandatory reporting in cases of cannabis ingestions, we hypothesize that reporting practices in our study population may be driven by clinical severity, perceived risk to the child, or evolving institutional norms. It is also plausible that societal and legal ambiguity surrounding cannabis, particularly in the transition from medical to recreational legalization, contributes to a lower threshold for reporting. As social acceptance and regulatory frameworks stabilize, these practices may evolve.

These findings carry important implications for both clinical care and public health policy. The frequent involvement of SW and CPS in our study, compared to variable rates in prior studies [2, 7, 8, 13], supports consideration of standardized pathways to guide social evaluation in cases of unintentional cannabis ingestion. This may help reduce institutional variation and support equitable decision-making. Notably, providers may consider the circumstances surrounding individual ingestion cases to determine if reporting is necessary, rather than universal mandatory reporting [37–39]. Given that most exposures occurred at home and were linked to edible products, integrating targeted developmentally appropriate safe-storage counseling into emergency and primary care encounters is essential; this should include emphasis on locked, hidden storage and non-appealing, child-resistant packaging. Collaboration between clinicians, public health agencies, and regulatory bodies is critical as cannabis products evolve, particularly with respect to packaging standards, clear dosing units, and restrictions on child-appealing formulations. Finally, the substantial proportion of children presenting with high acuity and requiring admissions suggest that pediatric emergency systems should anticipate continued resource demands related to cannabis ingestion and incorporate this trend into planning for staffing, training, and clinical protocols as legalization and product availability expand.

It is important to note that there are several limitations to this study. First, while we found no difference of sociodemographic factors on safe disposition planning, CPS evaluation continues beyond discharge and can vary by state and region; for example, this could mean an eventual home safety evaluation or placement back with a primary caregiver despite initial placement with a relative on discharge. It is unclear if these ultimate decisions are affected by sociodemographic factors. Additionally, families may call poison control for recommendations prior to hospital evaluation; local policies can vary in terms of poison control center mandatory reporting and recommendations for emergency evaluation. As such, some children with unintentional ingestions may be missed in this cohort if they did not present for PED care. While we have considered certain sociodemographic variables in our analysis, they may be other factors unaccounted for that affect the social management and disposition of these children, such as prior CPS involvement due to concerns of abuse or neglect. Additionally, some cases may be missed due to synthetic cannabinoid products, which may not prompt positive THC results on conventional UDS. Similarly, in patients presenting with a known ingestion, a UDS may not have been obtained and thus could be missing from the data set. There may also be data that is missing or incorrectly listed in the EMR. It is also noted that although the overall cohort exceeded 200 patients, several variables of interest, including non-English language status and absence of SW consultation, occurred infrequently. The resulting wide confidence intervals suggest limited power to detect potential associations. Future studies with larger samples are needed to further evaluate these relationships. Additionally, site-level clustering was not incorporated into the regression models. Because several outcomes were highly prevalent (e.g., social work consultation occurred in > 95% of cases) and the overall sample size was modest (*N* = 266), meaningful site-level variation was considered unlikely. However, residual cluster-level confounding related to unmeasured differences across sites cannot be excluded. Finally, this study was limited to two PED; these results may not be generalizable to the larger population.

## Conclusions

Cannabis ingestions pose a serious toxicity risk to young children; these ingestions are increasing in frequency. There is limited data on the influence of socioeconomic factors on management and safe disposition planning in these children. Analyzing cases from two large pediatric hospitals (2016–2024), we found that ingestions increased over time, primarily involved edibles, and often required urgent medical care, with high rates of hospital admission and social service involvement. Importantly, no significant association was found between socioeconomic status and social service engagement and disposition planning, suggesting decisions are driven by medical urgency rather than demographic factors. These findings highlight the growing burden on pediatric hospitals. Despite existing prevention efforts, the incidence of cannabis ingestions in young children continues to rise, suggesting that current strategies are insufficient. Future prevention efforts should be multifactorial and evidence-based, with a focus on primary prevention and ensuring equitable care for affected children and families.

**Table 1:** Descriptive characteristics of study population

Characteristic	*N* (%)
Race/Ethnicity	
Non-Hispanic White	115 (43.2)
Non-Hispanic Black	103 (38.7)
Hispanic	15 (5.6)
Other/Unknown*	33 (12.4)
Age	
< 1	27 (10.2)
1	72 (27.1)
2	56 (21.1)
3	47 (17.7)
4	30 (11.3)
5	19 (7.1)
6	15 (5.6)
Sex	
Male	139 (52.3)
Female	127 (47.7)
Language	
English	263 (98.9)
Spanish	1 (0.4)
Other**	2 (0.8)
Insurance	
Commercial	58 (21.8)
Public	190 (71.4)
Self-pay	0 (0)
Unknown	18 (6.8)
Location	
CCHMC (Main Campus)	147 (55.3)
CCHMC (Liberty Campus)	42 (15.8)
Nemours	77 (28.9)

*Other = American Indian or Alaska native, Asian, Native Hawaiian or Other Pacific Islander, other (not specified), two or more races

**Other = French, American Sign Language

**Table 2:** Medical care and disposition characteristics of study population

Characteristic	*N* (%)
Chief Complaint	
Altered Mental Status	108 (40.6)
Fatigue	6 (2.3)
Ingestion	100 (37.6)
Seizures	16 (6)
Vomiting	4 (1.5)
Well Child Care	4 (1.5)
Alleged Abuse	2 (0.8)
Other	26 (9.8)
ESI Level	
1	31 (11.7)
2	191 (71.8)
3	37 (13.9)
4	7 (2.6)
5	0 (0)
Trauma Resuscitation Evaluation	
Yes	111 (41.7)
No	255 (58.3)
Admit Service	
Hospital Floor	161 (60.5)
Critical Care	55 (20.7)
Psychiatric Care	1 (0.4)
Other*	1 (0.4)
Discharge from PED	48 (18)
Patient survival at discharge	
Yes	266 (100)
No	0 (0)

Other* = Surgery

**Table 3:** Ingestion characteristics of the study population

Characteristic	*N* (%)
Suspected Ingestion Type	
Flower	17 (6.4)
Edible	136 (51.1)
Vape	7 (2.6)
Oil	4 (1.5)
Other	7 (2.6)
Unknown	95 (35.7)
Suspected Source Owner	
Primary guardian	107 (40.2)
Extended family	49 (18.4)
Caregiver	4 (1.5)
Other	15 (5.6)
Unknown	91 (34.2)
Suspected Location of Ingestion	
Primary residence	169 (63.5)
Extended family residence	37 (13.9)
Daycare/School	3 (1.1)
Public Space	20 (7.5)
Other	8 (3.0)
Unknown	29 (10.9)
THC Confirmatory Testing	
Positive	253 (95.1)
Negative	11 (4.1)
Unknown	2 (0.8)

**Table 4:** Social management and disposition characteristics of the study population

Characteristic	*N* (%)
SW Consultation	
Yes	254 (95.5)
No	12 (4.5)
CPS Reporting	
Yes	213 (80.1)
No	53 (19.9)
Disposition Location following CPS Report	
Primary residence	226 (85.0)
Other*	37 (13.9)
Disposition Guardian following CPS Report	
Primary guardian	218 (82.6)
Other**	46 (17.3)

Other* = Relative non-primary guardian home, foster home placement, other/unknown

Other** = Relative non-primary guardian, foster placement, other/unknown

## Data Availability

All data is maintained at Cincinnati Children’s Hospital Medical Center and Nemours Children’s Health on password protected computers. The datasets generated and/or analyzed during the current study are not publicly available due containing identifying/contact information but are available from the corresponding author on reasonable request.

## Abbreviations

PED: Pediatric emergency department
SW: Social work
CPS: Child Protective Services
EMR: Electronic Medical Record
CCHMC: Cincinnati Children’s Hospital Medical Center
NCH: Nemours Children’s Hospital
THC: Tetrahydrocannabinol
UDS: Urine drug screen
ESI: Emergency Severity Index
SDI: Social deprivation index
B: Beta coefficient
CI: Confidence Interval
OR: Odds ratio
